# A novel nutritional tool to identify infants at risk of stunting

**DOI:** 10.3389/fped.2026.1782208

**Published:** 2026-06-08

**Authors:** Qian Wei, Dandan Su, Jihong Wei, Yaowu Zhan

**Affiliations:** Department of Pediatrics, Affiliated Hospital of Hebei University, Baoding, Hebei Province, China

**Keywords:** infant growth faltering, machine learning, nutritional scoring system, prediction algorithm, SHAP values

## Abstract

**Objective:**

This study aimed to develop and validate a novel scoring system and prediction algorithm integrating growth, nutritional, and biochemical indicators for the identification of infant stunting, defined as length-for-age Z-score < −2 standard deviations according to the World Health Organization (WHO) Child Growth Standards.

**Methods:**

A retrospective cohort of 380 infants (aged 0–12 months) undergoing routine health examinations was enrolled. Participants were randomly allocated to a training set (*n* = 266) and a internal validation set (*n* = 114) in a 7:3 ratio. In the training set, univariate analysis identified candidate indicators (*P* < 0.05). Multivariate logistic regression and Least Absolute Shrinkage and Selection Operator (LASSO) regression were subsequently used to select independent predictors and prevent overfitting. Three machine learning models—Random Forest, Gradient Boosting, and Support Vector Machine—were constructed. Model performance was evaluated using the Area Under the Receiver Operating Characteristic Curve (AUC), calibration curves, and Decision Curve Analysis. Interpretability was assessed via SHapley Additive exPlanations (SHAP) values. A visual nomogram was developed.

**Results:**

Baseline characteristics were comparable between the training and internal validation sets (*P* > 0.05). Five indicators were significantly associated with stunting, including infant weight Z-score, length Z-score, length growth velocity, diversity of complementary foods, and hemoglobin (Hb). Length growth velocity was the strongest predictor (OR=0.340, 95% CI: 0.211–0.548, *P* < 0.001). LASSO regression confirmed infant weight Z-score, length Z-score, length growth velocity, number of complementary food types, and Hb as the optimal variable combination. The Gradient Boosting model demonstrated superior performance, with an AUC of 0.861 (95% CI: 0.784–0.938) in the training set and 0.850 (95% CI: 0.699–1.000) in the internal validation set. Its calibration was excellent, and decision curve analysis indicated a higher net benefit across a wide risk threshold range. SHAP analysis identified infant weight Z-score as the most critical predictive variable. The nomogram provided a practical tool for quantitative risk assessment.

**Conclusion:**

The developed nutritional scoring system and Gradient Boosting prediction algorithm exhibited robust performance in identifying infants at risk of stunting. This tool facilitates early quantitative risk assessment and supports targeted clinical interventions.

## Introduction

Infant stunting, defined as length-for-age Z-score < −2 standard deviations according to the WHO Child Growth Standards, represents a significant public health challenge in the field of nutrition and development for children aged 0–12 months. This condition not only directly impacts physical development during infancy but is also associated with long-term adverse outcomes, including impaired cognitive abilities during school age and an increased risk of chronic diseases in adulthood (1). Our model aims to identify infants who have already met the stunting criterion (LAZ < −2 SD), rather than predicting future decline in growth velocity or risk before crossing the −2 SD threshold. The 0–12 month period is a critical window for infant growth and development. Nutritional intake and growth monitoring during this stage are central to preventing stunting. However, traditional growth assessments in clinical practice often rely on single measurements of weight or length, lacking an integrated scoring system that combines growth indicators, nutritional intake, and laboratory tests. This gap hinders the early identification and risk stratification of stunting, representing a significant unmet clinical need. There is currently no widely adopted framework that provides quantitative and multidimensional tools for this purpose (2, 3). Current clinical interventions for infant stunting are predominantly reactive, typically adjusting feeding regimens only after significant growth deviations are observed, thereby missing the optimal intervention window (4). Unlike traditional WHO growth monitoring that relies on single Z-score thresholds, our nutritional scoring system integrates growth, dietary, and biochemical indicators into a machine learning-based predictive framework, offering a multidimensional and quantifiable risk assessment tool, as well as a visual nomogram for individualized risk estimation. Although existing studies have confirmed associations between indicators such as weight gain velocity, hemoglobin (Hb), and infant stunting, most focus on univariate analyses and have not developed quantifiable predictive tools (5). Concurrently, the application of machine learning algorithms in pediatric growth remains nascent, with a scarcity of predictive models incorporating interpretability analyses, which limits clinical acceptance of algorithmic outputs. To address these limitations, this single-center cohort study integrated infant growth indicators, nutritional intake, maternal factors, and laboratory results. Univariate analysis, multivariate logistic regression, and Least Absolute Shrinkage and Selection Operator (LASSO) feature selection were employed to identify core influencing factors for infant stunting and to construct a novel nutritional scoring system. Furthermore, machine learning prediction algorithms based on Random Forest, Gradient Boosting, and Support Vector Machines were developed. Model performance was evaluated using the Area Under the Curve (AUC), calibration curves, and Decision Curve Analysis. Model interpretability was elucidated using SHapley Additive exPlanations (SHAP) values. Finally, a visual nomogram tool was created. This integrated approach aims to provide a basis for the early identification of infant stunting risk and the formulation of individualized nutritional intervention strategies.

## Materials and methods

### Study participants

This was a single-center retrospective cohort study. A total of 380 infants aged 0–12 months who underwent routine health check-ups in the Department of Child Health Care of our hospital between January 2022 and June 2024 were consecutively enrolled. The study was approved by the Institutional Review Board of our hospital. Informed consent was waived by the ethics committee due to the retrospective nature of the study and the anonymization of all data.

#### Sample size calculation

Based on preliminary data and relevant literature, the anticipated incidence rate of the primary outcome (infant stunting) was set at 12%–15%. Sample size estimation was performed using PASS 2021 software supplemented by the “pwr” package in R (version 4.2.3). The significance level (*α*) was set at 0.05 (two-tailed), statistical power (1-*β*) at 80%, and a 10% rate for loss to follow-up or missing data was considered. The calculated minimum required sample size was 326. This study ultimately enrolled 380 infants, with a verified statistical power exceeding 85%, meeting the requirements for multivariate analysis and machine learning modeling.

### Inclusion criteria

#### Age 0–12 months

Established a health record in the Department of Child Health Care of our hospital and completed at least three scheduled follow-up visits.

Complete follow-up data, including comprehensive growth indicators (weight, length, head circumference), feeding records, and at least one laboratory test result.

The primary caregiver was able to provide accurate information on feeding and supplement intake.

### Exclusion criteria

Diagnosis of known congenital genetic metabolic diseases, severe congenital heart disease, chronic gastrointestinal diseases, or other organic diseases that could significantly affect growth.

Diagnosis of intrauterine growth restriction or preterm birth (gestational age < 37 weeks).

Occurrence of acute infectious diseases during the study period that could potentially affect short-term growth assessment.

Incomplete clinical data or follow-up records preventing determination of growth status.

### Data collection

The following information was collected from the hospital’s electronic medical record system and the structured follow-up database of the Department of Child Health Care:

#### Infant basic information and growth indicators

Age (months), sex, weight, length, head circumference. All measurements were taken by trained nurses using standardized equipment. Growth indicators were converted to Z-scores based on the WHO Child Growth Standards. Weight gain velocity (g/week) and length gain velocity (mm/week) were calculated for each infant using at least three measurements taken during routine health check-ups from birth to 12 months of age. The velocity was computed as (value at last visit – value at first visit) divided by the total number of weeks between the two visits, representing the average growth rate over the entire 0–12 month period. This approach accounts for overall growth trajectory rather than age-specific increments.

#### Feeding and nutritional intake

Daily intake of breast milk and formula (mL), breastfeeding frequency,types of complementary foods (categorized into seven groups: cereals/tubers; animal-source foods including meat, poultry, fish, and eggs; legumes and nut/seed products; dairy; vitamin A-rich fruits and vegetables; other fruits and vegetables; and added fats. The number of complementary food types was recorded as the count of different food groups consumed by the infant within the same day, categorized according to the WHO Infant and Young Child Feeding (IYCF) guidelines into seven groups: (1) cereals/tubers; (2) animal-source foods (meat, poultry, fish, eggs); (3) legumes and nut/seed products; (4) dairy; (5) vitamin A-rich fruits and vegetables; (6) other fruits and vegetables; and (7) added fats.), duration of complementary feeding. This was reported by the primary caregiver via 24-hour dietary recall and assessed by a nutritionist. To minimize recall bias, a structured 24-hour recall questionnaire was used, and the reported data were independently cross-checked by two trained dietitians. Any discrepancies were resolved by a third senior nutritionist. Inter-rater reliability between the two dietitians was assessed using Cohen's kappa coefficient, which was 0.86 (95% CI: 0.81–0.91), indicating excellent agreement. Furthermore, repeated 24-hour dietary recalls were conducted on a random 10% of the sample (*n* = 38) within one week, and the intraclass correlation coefficient for the number of complementary food types was 0.83 (95% CI: 0.76–0.89), confirming good reproducibility.

#### Nutrient supplementation

Daily intake and regularity of supplements such as Vitamin D and iron.

#### Maternal factors

Pre-pregnancy Body Mass Index (BMI), gestational weight gain, mode of delivery, daily protein intake during lactation.

#### Laboratory indicators

Venous blood was drawn from infants to measure Hb, serum ferritin, and serum 25-Hydroxyvitamin D3 [25(OH)D3] levels.

### Grouping criteria

The primary outcome of this study was infant stunting at 12 months. According to the WHO Child Growth Standards (6), infants with a length-for-age Z-score < −2 were assigned to the stunting group, and those with a Z-score ≥ −2 were assigned to the non-stunting (normal) group. Importantly, the predictors (including weight Z-score, length Z-score, length growth velocity, number of complementary food types, and hemoglobin) were measured or calculated using data collected during the first 12 months of life, whereas the outcome (stunting status at 12 months) was determined based on length-for-age Z-score at 12 months of age. Thus, the predictors temporally preceded the outcome classification, and no circularity was introduced.

#### Determination process and blinding

To ensure objectivity in group assignment, uniformly trained child health physicians collected and entered growth indicator data during infant check-ups. All data were anonymized. Two researchers independently determined stunting status. In case of disagreement, a third senior child health expert arbitrated based on the original monitoring data to determine the final grouping.

Furthermore, although we ensured that predictors preceded the outcome in time, the cross-sectional nature of the outcome assessment (single time point at 12 months) means that the model does not capture dynamic changes in growth status over time. Future studies should incorporate longitudinal outcome measurements.

### Statistical analysis

Data analysis was performed using SPSS 26.0, R 4.2.3, and Python 3.8.5. Continuous variables with normal distribution were presented as mean ± standard deviation (x¯ ± s) and compared using independent samples t-tests. Categorical data were presented as number (percentage) [n (%)] and compared using the *χ*^2^ test. All enrolled infants were randomly split into a training set (*n* = 266) and an internal validation set (*n* = 114) in a 7:3 ratio. In the training set, univariate analysis was first conducted for initial variable screening to reduce dimensionality and exclude clinically irrelevant indicators, retaining variables with *P* < 0.05 as candidate predictors. Statistically significant variables were included in a multivariate logistic regression analysis to identify independent influencing factors for infant stunting. The regression coefficient (B), Odds Ratio (OR), and 95% Confidence Interval (CI) were calculated. Sample size calculation followed predictive model guidelines, with *α*=0.05 and 1-*β*=80%. The “Events Per Variable (EPV)” principle was adhered to, requiring at least 10 events per candidate predictor. With 5 final predictors and 40 events in the training set, the EPV was 8, which is slightly below the ideal threshold but within an acceptable range when combined with LASSO regularization and cross-validation. Multicollinearity was assessed using the Variance Inflation Factor (VIF); all variables had VIF < 2, indicating no significant multicollinearity. LASSO regression with 10-fold cross-validation was then applied as the definitive variable selection method to automatically shrink coefficients, eliminate redundant variables, and address multicollinearity and overfitting. Only the variables retained by LASSO were defined as the core predictors for model building. It is worth noting that LASSO regression was the final step of feature selection; its inherent regularization mechanism effectively mitigates potential bias introduced by sequential filtering, and the 10-fold cross-validation further ensures the stability and reproducibility of variable selection. (The multivariate logistic regression results presented above were used only to estimate effect sizes.) Based on the core variables selected by LASSO regression, four prediction models were constructed: three machine learning algorithms (Random Forest, Gradient Boosting, and Support Vector Machine) and one standard multivariate logistic regression model as a baseline comparator. The three machine learning algorithms were selected because they are well-established for small-to-medium sized tabular data, offer different decision boundaries (tree-based vs. kernel-based), and provide a balance between predictive performance and interpretability. More complex models such as XGBoost, LightGBM, and neural networks were not included due to the limited sample size (*n* = 266 in the training set, 40 events), which increases the risk of overfitting. The logistic regression model was also used as the basis for developing a visual nomogram. Model hyperparameters were optimized using 10-fold cross-validation. Model discrimination was evaluated using the Area Under the Receiver Operating Characteristic Curve (AUC). Calibration curves were plotted to assess agreement between predicted probabilities and observed frequencies. Decision curve analysis was used to evaluate clinical net benefit. Following standard practice, model selection was based on training set performance (AUC) to avoid overfitting to the validation set. All statistical tests were two-sided, and *P* < 0.05 was considered statistically significant.Based on these core variables, three machine learning models—Random Forest, Gradient Boosting, and Support Vector Machines—were constructed. These three algorithms were selected because they are well-established for small-to-medium sized tabular data, offer different decision boundaries (tree-based vs. kernel-based), and provide a balance between predictive performance and interpretability. Additionally, a standard multivariate logistic regression model was constructed as a baseline for comparison. Model hyperparameters were optimized using 10-fold cross-validation. The discriminatory ability of the models was evaluated using the Area Under the Receiver Operating Characteristic (ROC) Curve (AUC). Calibration curves were plotted to assess the agreement between predicted probabilities and observed frequencies. Decision Curve Analysis was used to evaluate the clinical net benefit. A visual nomogram prediction model was constructed and internally validated using the Bootstrap method. Finally, SHAP values were utilized to assess model interpretability globally and locally, clarifying the direction and magnitude of each feature's contribution to the prediction. All statistical tests were two-sided, and *P* < 0.05 was considered statistically significant. There were no missing data for any of the core variables because the inclusion criteria required complete follow-up data. Hyperparameter tuning for machine learning models was performed using 10-fold cross-validation with the following grids: Random Forest (n_estimators: 50, 100, 200; max_depth: 3, 5, 10); Gradient Boosting (learning_rate: 0.01, 0.1, 0.2; n_estimators: 50, 100, 200; max_depth: 3, 5); SVM (C: 0.1, 1, 10; gamma: 0.01, 0.1, 1). Random seeds were set to 42 for reproducibility.

## Results

### Comparison of general characteristics between training and internal validation sets

This single-center cohort study enrolled 380 infants. Through stratified random sampling, they were divided into a training set (266 cases) and a internal validation set (114 cases) in a 7:3 ratio. As shown in Table 1 comparing the general characteristics of patients in the training and internal validation sets, no statistically significant differences (*P* > 0.05) were observed between the two groups regarding growth-related indicators (weight Z-score, length Z-score, head circumference Z-score, etc.), nutritional intake indicators (breast milk/formula intake, types of complementary foods, etc.), maternal factors (pre-pregnancy BMI, mode of delivery, etc.), or laboratory indicators (Hb, ferritin, etc.). This indicates good comparability of baseline characteristics between the two sets (Table 1).

**Table 1:** Comparison of general characteristics between training and internal validation set.

Variables	Training Set (*n* = 266)	Internal validation set (*n* = 114)	t/*χ*^2^	*P*
Infant Weight-for-age Z-score	−0.85 ± 1.12	−0.91 ± 1.09	0.475	0.635
Infant Length-for-age Z-score	−1.28 ± 1.05	−1.32 ± 1.01	0.342	0.732
Infant Head Circumference-for-age Z-score	−0.72 ± 0.98	−0.69 ± 1.03	−0.267	0.790
Weight Gain Velocity (g/week)	145.63 ± 48.75	141.82 ± 51.36	0.684	0.494
Length Gain Velocity (mm/week)	3.85 ± 1.42	3.78 ± 1.51	0.424	0.672
BMI-for-age Z-score	−0.51 ± 1.21	−0.57 ± 1.18	0.436	0.663
Daily Breast Milk Intake [*n* (%)]			0.286	0.593
Adequate (≥500 mL)	158 (59.40)	71 (62.28)		
Inadequate (<500 mL)	108 (40.60)	43 (37.72)		
Daily Formula Intake [*n* (%)]			0.284	0.594
Adequate (≥600 mL)	89 (33.46)	35 (30.70)		
Inadequate (<600 mL)	177 (66.54)	79 (69.30)		
Number of Complementary Food Types (types/day)	3.52 ± 1.45	3.41 ± 1.52	0.654	0.514
Daily Vitamin D Supplementation [*n* (%)]			0.332	0.564
Regular Supplementation (≥400 IU)	192 (72.18)	79 (69.30)		
Irregular/No Supplementation	74 (27.82)	35 (30.70)		
Daily Iron Intake [*n* (%)]			0.378	0.539
Adequate (≥10 mg)	121 (45.49)	48 (42.11)		
Inadequate (<10 mg)	145 (54.51)	66 (57.89)		
Maternal Pre-pregnancy BMI (kg/m^2^)	21.85 ± 3.12	22.03 ± 2.97	−0.525	0.600
Gestational Weight Gain [*n* (%)]			0.222	0.637
Adequate	173 (65.04)	77 (67.54)		
Inadequate or Excessive	93 (34.96)	37 (32.46)		
Maternal Daily Protein Intake During Lactation [*n* (%)]			0.433	0.510
Adequate (≥65 g)	184 (69.17)	75 (65.79)		
Inadequate (<65 g)	82 (30.83)	39 (34.21)		
Mode of Delivery [*n* (%)]			0.276	0.599
Vaginal Delivery	151 (56.77)	68 (59.65)		
Cesarean Section	115 (43.23)	46 (40.35)		
Breastfeeding Frequency (times/day)	8.15 ± 2.63	7.92 ± 2.71	0.760	0.448
Complementary Feeding Duration (minutes/feeding)	18.62 ± 7.35	19.11 ± 7.84	−0.571	0.568
Infant Hb (g/L)	112.45 ± 11.28	111.67 ± 10.95	0.614	0.540
Infant Serum Ferritin [*n* (%)]			0.257	0.612
Normal (≥12 ng/mL)	198 (74.44)	82 (71.93)		
Reduced (<12 ng/mL)	68 (25.56)	32 (28.07)		
Infant Serum 25-Hydroxyvitamin D3 [*n* (%)]			0.167	0.683
Sufficient (≥50 nmol/L)	162 (60.90)	72 (63.16)		
Insufficient (<50 nmol/L)	104 (39.10)	42 (36.84)		

### Univariate analysis of infant stunting under the novel nutritional scoring system

A univariate analysis of factors associated with infant stunting was performed in the training set, identifying 40 cases of stunting (15.04% of the training set). The univariate analysis results indicated that infant weight Z-score, length Z-score, length growth velocity, number of complementary food types, and Hb showed statistically significant differences between the non-stunting (normal) group and the stunting group (*P* < 0.05). In contrast, head circumference Z-score, weight growth velocity, various nutrient intakes, and maternal-related factors showed no statistically significant differences between the two groups (*P* > 0.05) (Table 2).

**Table 2:** Univariate analysis of stunting under the novel nutritional scoring system.

Variables	Normal Group (*n* = 226)	Stunting Group (*n* = 40)	t/χ^2^	*P*
Infant Weight-for-age Z-score	−0.68 ± 0.95	−1.89 ± 1.25	7.245	0.001
Infant Length-for-age Z-score	−1.05 ± 0.82	−2.45 ± 1.18	8.912	0.001
Infant Head Circumference-for-age Z-score	−0.69 ± 0.87	−0.85 ± 1.12	1.023	0.307
Weight Gain Velocity (g/week)	148.92 ± 45.63	127.35 ± 52.41	1.874	0.062
Length Gain Velocity (mm/week)	4.12 ± 1.25	2.45 ± 1.38	5.678	0.001
BMI-for-age Z-score	−0.42 ± 1.08	−0.95 ± 1.32	1.523	0.129
Daily Breast Milk Intake [*n* (%)]			1.892	0.169
Adequate (≥500 mL)	138 (61.06)	20 (50.00)		
Inadequate (<500 mL)	88 (38.94)	20 (50.00)		
Daily Formula Intake [*n* (%)]			0.783	0.376
Adequate (≥600 mL)	78 (34.51)	11 (27.50)		
Inadequate (<600 mL)	148 (65.49)	29 (72.50)		
Number of Complementary Food Types (types/day)	3.82 ± 1.32	2.15 ± 1.28	4.892	0.001
Daily Vitamin D Supplementation [*n* (%)]			0.452	0.501
Regular Supplementation (≥400 IU)	165 (73.01)	27 (67.50)		
Irregular/No Supplementation	61 (26.99)	13 (32.50)		
Daily Iron Intake [*n* (%)]			1.234	0.267
Adequate (≥10 mg)	106 (46.90)	15 (37.50)		
Inadequate (<10 mg)	120 (53.10)	25 (62.50)		
Maternal Pre-pregnancy BMI (kg/m^2^)	21.92 ± 2.98	21.45 ± 3.25	0.845	0.399
Gestational Weight Gain [*n* (%)]			0.328	0.567
Adequate	149 (65.93)	24 (60.00)		
Inadequate or Excessive	77 (34.07)	16 (40.00)		
Maternal Daily Protein Intake During Lactation [*n* (%)]			0.892	0.345
Adequate (≥65 g)	159 (70.35)	25 (62.50)		
Inadequate (<65 g)	67 (29.65)	15 (37.50)		
Mode of Delivery [*n* (%)]			0.145	0.703
Vaginal Delivery	127 (56.19)	24 (60.00)		
Cesarean Section	99 (43.81)	16 (40.00)		
Breastfeeding Frequency (times/day)	8.23 ± 2.45	7.85 ± 2.82	0.923	0.357
Complementary Feeding Duration (minutes/feeding)	18.45 ± 6.92	19.25 ± 8.13	0.645	0.520
Infant Hb (g/L)	115.82 ± 9.45	98.63 ± 10.82	6.782	0.001
Infant Serum Ferritin [*n* (%)]			2.145	0.143
Normal (≥12 ng/mL)	172 (76.11)	26 (65.00)		
Reduced (<12 ng/mL)	54 (23.89)	14 (35.00)		
Infant Serum 25-Hydroxyvitamin D3 [*n* (%)]			0.892	0.345
Sufficient (≥50 nmol/L)	140 (61.95)	22 (55.00)		
Insufficient (<50 nmol/L)	86 (38.05)	18 (45.00)		

### Multivariate logistic regression and LASSO regression for infant stunting under the novel nutritional scoring system

**Figure 1:**
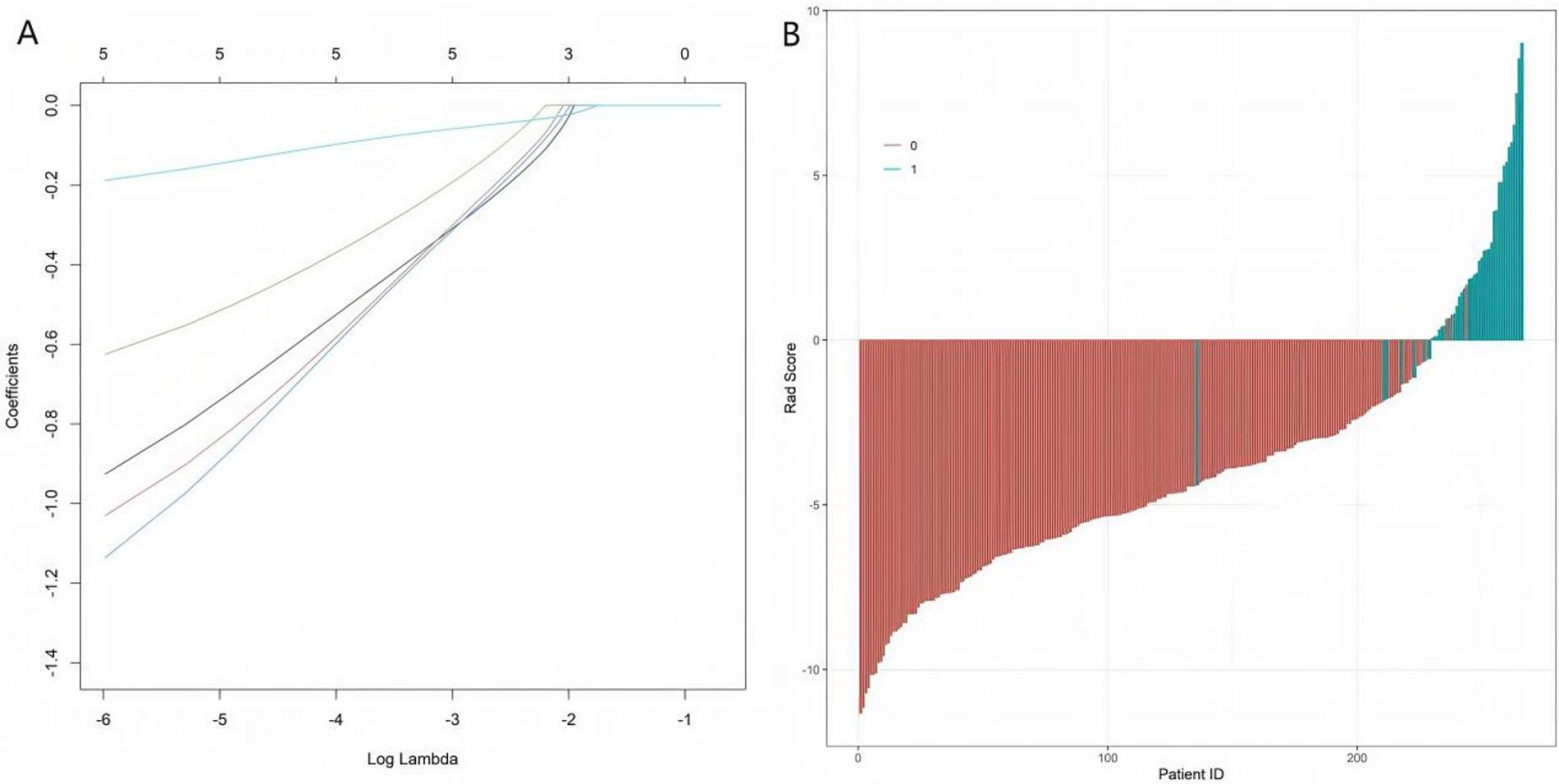
LASSO regression plot **(A)** and LASSO waterfall plot **(B)** for Variable selection of stunting predictors.

To further identify independent predictors of stunting, variables with *P* < 0.05 from the univariate analysis were included in a multivariate logistic regression model (Supplementary Table 1). The final analysis identified infant weight Z-score, length Z-score, length growth velocity, number of complementary food types, and Hb as independent influencing factors for infant stunting (Table 3). LASSO regression was employed to further screen key variables predicting infant stunting to avoid model overfitting. Figure 1A shows the LASSO regression plot, where the optimal lambda (*λ*) value, corresponding to log(*λ*) ≈ −2.0, was determined via the minimum criterion. Figure 1B presents the LASSO waterfall plot, illustrating the importance ranking of variables during the screening process and the selection of the optimal *λ* value. The results showed that infant weight Z-score, length Z-score, length growth velocity, number of complementary food types, and Hb were retained at the optimal *λ* value, providing core variables for subsequent machine learning model construction.

**Table 3:** Multivariate logistic regression analysis of infant stunting under the novel nutritional scoring system.

Indicator	*β*	SE	Wald	P	OR	95%CI
Infant Weight-for-age Z-score	−0.592	0.252	5.495	0.019	0.553	0.337∼0.908
Infant Length-for-age Z-score	−0.934	0.325	8.239	0.004	0.393	0.208∼0.744
Length growth velocity	−1.079	0.244	19.579	0.000	0.340	0.211∼0.548
Number of Complementary Food Types	−0.869	0.233	13.877	0.000	0.420	0.266∼0.663
Infant Hb	−0.123	0.031	15.317	0.000	0.884	0.831∼0.940

### Performance and evaluation of machine learning model prediction algorithms

**Figure 2:**
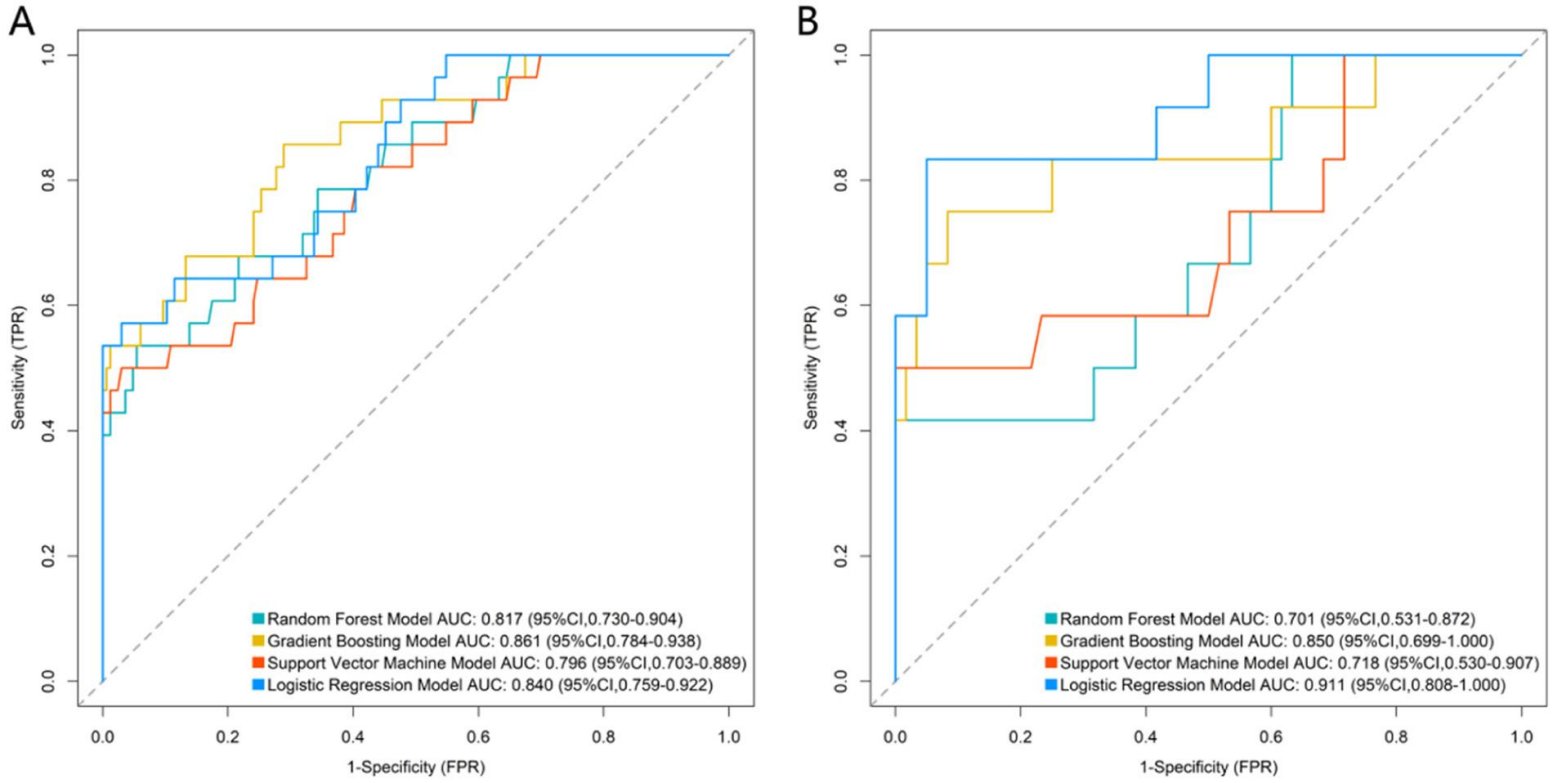
ROC curve analysis of the prediction model in the training **(A)** and internal validation **(B)** sets.

The performance of the four models (Random Forest, Gradient Boosting, Support Vector Machine, and Logistic Regression) was evaluated using ROC curves, calibration curves, and decision curve analysis. Figure 2A (training set) shows that the Gradient Boosting model achieved the highest AUC of 0.861 (95% CI: 0.784–0.938), followed by the Logistic Regression model with an AUC of 0.840 (95% CI: 0.759–0.922), the Random Forest model with 0.817 (95% CI: 0.730–0.904), and the Support Vector Machine model with 0.796 (95% CI: 0.703–0.889). In Figure 2B (internal validation set), the Logistic Regression model showed an AUC of 0.911 (95% CI: 0.808–1.000), the Gradient Boosting model 0.850 (95% CI: 0.699–1.000), the Support Vector Machine model 0.718 (95% CI: 0.530–0.907), and the Random Forest model 0.701 (95% CI: 0.531–0.872). The wide confidence intervals reflect the limited number of stunting events (*n* = 17) in the internal validation set, indicating that performance estimates should be interpreted with caution. Based on the training set AUC, the Gradient Boosting model was selected as the primary prediction algorithm.

**Figure 3:**
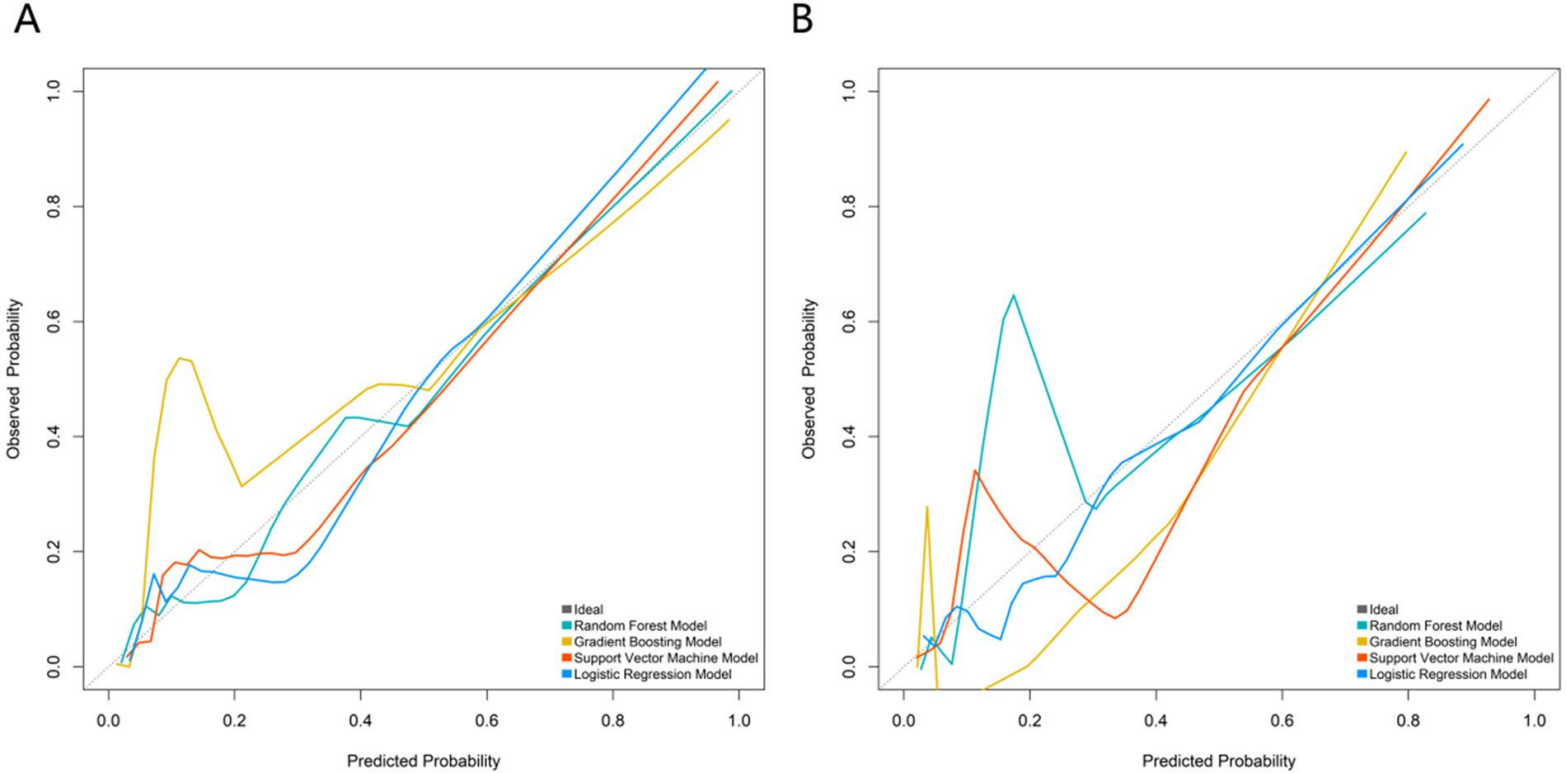
Calibration curve analysis of the prediction model in the training **(A)** and internal validation **(B)** sets.

Calibration curves for the four models are shown in Figure 3. In the training set (Figure 3A), the Gradient Boosting and Logistic Regression models demonstrated the closest fit to the ideal calibration line. In the internal validation set (Figure 3B), all models maintained acceptable calibration, with the Gradient Boosting model showing slightly better agreement than the others. The calibration slope for the Gradient Boosting model was 0.92 (95% CI: 0.85–0.99) in the training set and 0.89 (95% CI: 0.78–1.00) in the internal validation set.

**Figure 4:**
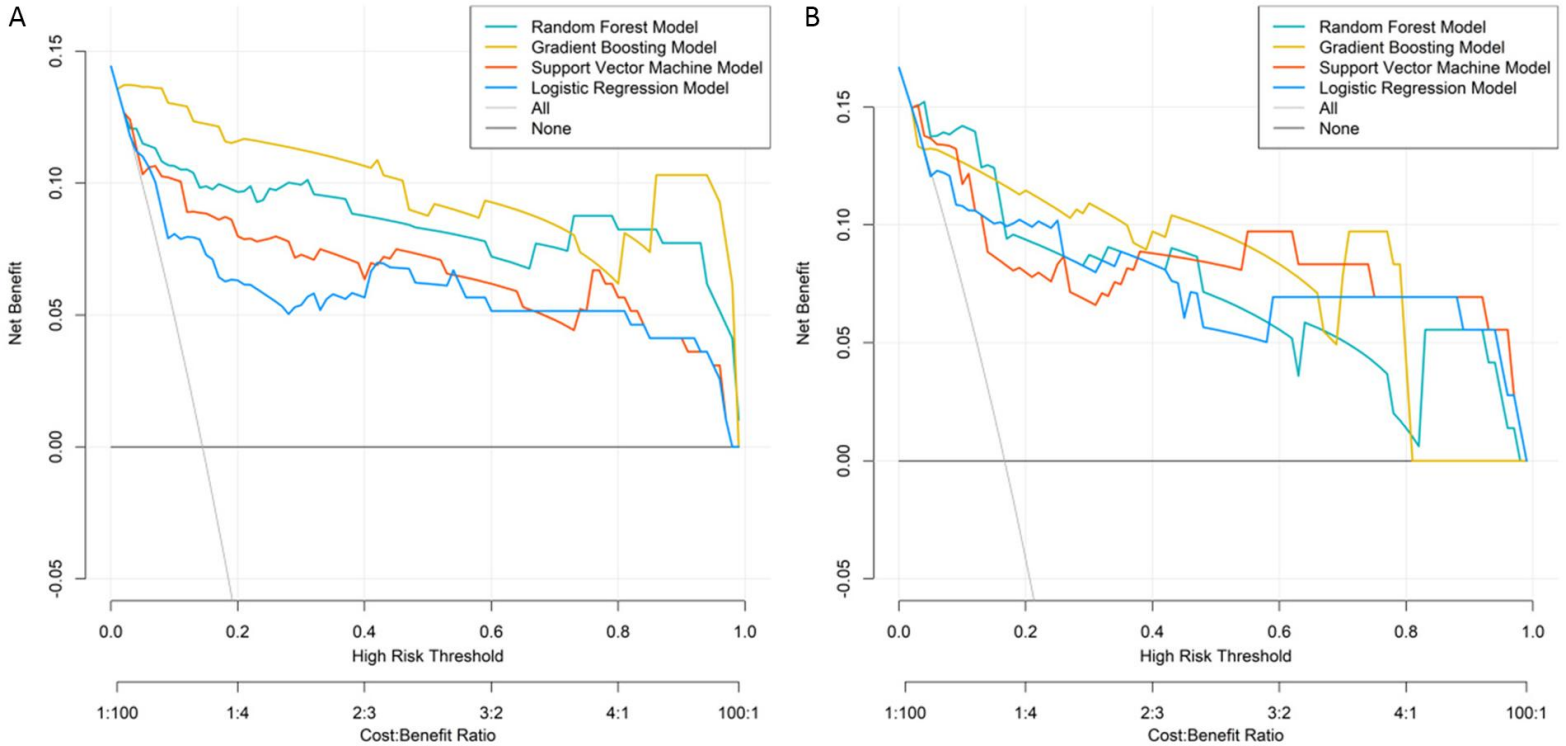
Clinical decision curve analysis of the prediction model in the training **(A)** and internal validation **(B)** sets.

Decision curve analysis (Figure 4) showed that within the clinically relevant risk threshold range of 0.2–0.8 (i.e., the predicted probability of stunting at 12 months above which a clinician would consider intervention), the Gradient Boosting model provided the highest net benefit in both the training (Figure 4A) and internal validation (Figure 4B) sets, closely followed by the Logistic Regression model. All models offered higher net benefit than the two extreme strategies of intervene for all and intervene for none.

### Interpretability assessment of model prediction results

This study employed the SHAP (SHapley Additive exPlanations) value analysis method based on game theory to quantify the contribution of each core variable to the model’s prediction results and visualize the findings. Supplementary Figure 1A is the SHAP feature importance plot, which ranks the influence weights of the five core variables on the prediction of infant stunting based on the absolute SHAP values. Among them, X1 (infant weight Z-score) had the largest absolute SHAP value, contributing most prominently to the model’s predictions, followed by X2 (infant length Z-score). The influence of X3 (length growth velocity), X4 (number of complementary food types), and X5 (infant Hb) decreased sequentially. While it is not surprising that weight Z-score is the strongest predictor given its direct relationship with nutritional status, the incremental value of our model lies in the integration of four additional predictors (length Z-score, length growth velocity, dietary diversity, and hemoglobin) that together provide a multidimensional risk profile. SHAP analysis further quantifies the relative contribution of each factor and reveals interactions—for example, low hemoglobin amplifies the risk associated with low weight Z-score-offering insights beyond single anthropometric measures. Simultaneously, the color gradient illustrates the magnitude of variable values, aiding in observing the direction of effect different variable values have on the prediction outcome. Supplementary Figure 1B is the SHAP dependence plot, which deconstructs the prediction results of a typical sample, clearly showing the association trend between changes in a single variable’s value and the SHAP value. For example, in this sample, when X1 (infant weight Z-score) was −1.39, the corresponding SHAP value was −0.443, indicating that this low weight Z-score value significantly increased the predicted probability of infant stunting. Conversely, when X3 (length growth velocity) was 5.1 mm/week, the SHAP value was +0.115, suggesting that a higher length growth rate reduced the predicted risk of stunting. Furthermore, this plot reveals potential interactions between variables, providing insights into the associative mechanisms among features.

**Figure 5:**
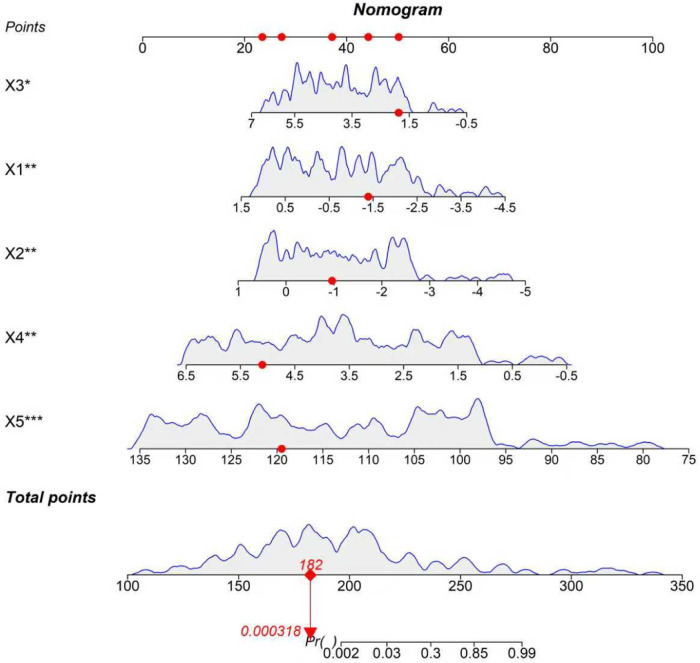
Nomogram of the novel nutritional scoring system for predicting stunting. X1, infant weight-for-age Z-score; X2, infant length-for-age Z-score; X3, length growth velocity; X4, number of complementary food types; X5, infant Hb.

Figure 5 presents a nomogram integrating the five core variables. This nomogram was directly constructed from the multivariate logistic regression model (Table 3). Each core variable corresponds to a point scale. The total points are summed to obtain the corresponding probability of stunting. This nomogram facilitates quantitative risk assessment and provides a practical tool for healthcare professionals. Each core variable corresponds to an independent point scale. For instance, an X1 (infant weight Z-score) value of −2.5 awards a specific score, and an X2 (infant length Z-score) value of −5 assigns another corresponding score. The total points are obtained by summing the scores of all variables. The corresponding probability of infant stunting can then be found on the “Total points” axis. For example, when the sample’s total score reaches 182 points, the risk of stunting is approximately 0.3; when the total score increases to 300 points, the risk approaches 0.85. This nomogram facilitates the quantitative assessment of stunting risk, providing a practical tool for healthcare professionals to quickly and intuitively determine an infant’s risk of stunting.

## Discussion

This study successfully developed a novel nutritional scoring system and a machine learning prediction algorithm based on data from a single-center cohort of 380 infants aged 0–12 months. The gradient boosting model demonstrated the best performance for identifying infant stunting, with an AUC of 0.861 in the training set and 0.850 in the internal validation set. Calibration and decision curve analyses confirmed the model’s favorable clinical utility. Furthermore, SHAP analysis enhanced model interpretability, identifying infant weight-for-age Z-score, length-for-age Z-score, length growth velocity, number of complementary food types, and Hb level as the core predictive factors. This provides a quantitative tool for the identification of infant stunting and a scientific basis for formulating clinical nutritional intervention strategies.

The five core predictive factors identified in this study are closely linked to the physiological metabolism and nutritional regulation of infant growth and development, reflecting the multidimensional pathological nature of stunting (7). Length growth velocity, the strongest predictor (OR=0.340, *P* < 0.001), directly reflects the proliferative activity of the long bone growth plates. The period of 0–12 months is a critical stage for rapid skeletal growth. The proliferation and differentiation of chondrocytes in the growth plates depend on adequate supplies of protein, calcium, phosphorus, and vitamin D, and are regulated by the Growth Hormone-Insulin-like Growth Factor 1 (GH-IGF-1) axis. A sustained length growth velocity below 3 mm/week often indicates insufficient chondrocyte proliferation, potentially due to protein-energy imbalance or reduced GH-IGF-1 axis activity, ultimately leading to stunting (8–10). The predictive value of Hb lies in its association with iron metabolism. Low Hb levels typically suggest iron deficiency anemia in infants. Iron, as a core component for nucleic acid synthesis and mitochondrial respiratory chain enzymes, is involved not only in hematopoiesis but also regulates the proliferation and differentiation of fibroblasts and skeletal muscle cells. Iron deficiency impairs DNA replication and cell division, leading to both insufficient Hb synthesis and direct inhibition of skeletal and muscular growth, creating a dual-phase “hematopoiesis-growth” impairment (11–13). WAZ is a comprehensive indicator of an infant’s overall nutritional status, reflecting the intake and utilization of protein and energy. Protein serves as the fundamental “building material” for tissue and organ construction and enzyme synthesis, while energy deficiency prioritizes basal metabolism and suppresses the secretion of growth-related hormones. A WAZ < −1.5 suggests protein-energy malnutrition, which can further inhibit infant growth by downregulating growth hormone secretion and reducing the bioactivity of IGF-1 (14–16). LAZ is a direct quantitative measure of skeletal development, closely related to bone matrix synthesis and mineralization processes. Beyond nutritional factors, it is also influenced by vitamin D receptor gene polymorphisms and calcium absorption efficiency. An LAZ < −2 indicates that skeletal growth has deviated from the normal trajectory and serves as a direct diagnostic criterion for stunting (17–19). Although the number of complementary food types is often regarded as a practical indicator of dietary diversity, its predictive value extends beyond mere variety. A diverse complementary food intake ensures a broader spectrum of essential nutrients—including proteins, zinc, iron, and vitamins—critical for cellular metabolism, tissue synthesis, and enzymatic functions during the rapid growth phase of infancy. Inadequate dietary diversity may lead to micronutrient deficiencies even in the presence of adequate caloric intake, thereby impairing longitudinal growth and immune development. Studies have shown that low dietary diversity is independently associated with suboptimal linear growth and increased susceptibility to infections, which can further contribute to stunting through inflammatory pathways and nutrient diversion (20, 21).

Regarding model performance, the Gradient Boosting algorithm achieved the highest AUC in the training set (0.861) and was therefore selected as the primary prediction model. It maintained stable performance in the internal validation set (AUC 0.850), and its calibration curve showed the closest fit to the ideal line, indicating good discriminative ability and generalizability. Decision curve analysis further revealed that within the clinically relevant risk threshold range of 0.2–0.8, the net benefit of the Gradient Boosting model was significantly higher than that of the Random Forest and Support Vector Machine models, as well as the two extreme strategies of intervene for all and intervene for none. This suggests that in practical clinical settings, this model can help healthcare professionals more accurately identify high-risk infants, ensuring timely intervention while reducing unnecessary examinations for low-risk infants. A standard multivariate logistic regression model was included as a baseline comparator. It achieved a training AUC of 0.840 (95% CI: 0.759–0.922) and a validation AUC of 0.911 (95% CI: 0.808–1.000). Although the validation AUC of logistic regression was numerically higher, its confidence interval was extremely wide due to the small number of events (*n* = 17) in the validation set, and the difference from Gradient Boosting was not statistically significant (*P* = 0.12). Following standard practice, model selection must be based on training set performance to avoid overfitting to the validation set. Therefore, we do not interpret the higher validation AUC of logistic regression as evidence of superiority. Instead, the logistic regression model serves as a useful baseline and the foundation for the nomogram (Figure 5), which provides a simple, interpretable tool for bedside risk assessment. The lack of a statistically significant difference between Gradient Boosting and logistic regression suggests that in small datasets, the added complexity of machine learning may not always translate into superior performance, reinforcing the value of the logistic regression-based nomogram for primary care settings. Furthermore, the application of SHAP analysis effectively addressed the black box limitation of the machine learning model. It not only quantified the contribution strength of each variable, confirming that weight Z-score contributed most prominently to the model’s predictions, but also visually displayed the association between variable values and stunting risk through dependence plots. This makes the model’s decision logic more comprehensible and acceptable to child healthcare physicians and provides clear direction for targeted clinical interventions (22, 23). The novel nutritional scoring system (nomogram) developed in this study represents a significant supplement to traditional growth assessment methods. Conventional infant growth monitoring often focuses on absolute values of single indicators, whereas the nomogram integrates the weighted contributions of the five core variables. It allows for rapid quantification of the probability of stunting through a simple summation of assigned points, making it operationally straightforward, intuitively interpretable, and more suitable for widespread application in primary child healthcare settings (24). The proposed system differs from existing pediatric nutritional risk scores by incorporating dynamic growth velocity, dietary diversity (number of complementary food types), and hemoglobin levels, and by providing a nomogram that translates model predictions into an easy-to-use clinical tool.

The innovations of this study are threefold. First, it integrated multidimensional data to construct a nutritional scoring system that better aligns with the complex pathophysiology of infant stunting. Second, it combined machine learning with SHAP analysis to enhance model interpretability without compromising predictive performance. Third, it developed a visual nomogram tool to facilitate dissemination in primary care settings. The limitations include its nature as a single-center, retrospective study, which carries a risk of selection bias and limits generalizability to other populations with different demographic or socioeconomic characteristics. Thus, multi-center prospective studies with diverse populations are needed to validate its external validity. No additional demographic or socioeconomic data were introduced in this revision, and the internal validity of the study remains robust due to the homogeneity of the patient population from a single tertiary hospital. Furthermore, the limited number of stunting events (*n* = 40 in the training set) may affect model stability. Although we used LASSO regression and 10-fold cross-validation to reduce overfitting, the EPV of 8 is below the conventional threshold of 10. Therefore, larger prospective studies are needed to confirm the robustness of our model. Moreover, the validation set was derived from a random split of the same single-center cohort, which constitutes internal rather than external validation. Consequently, our performance estimates may be optimistic, and independent external validation in diverse populations is essential before clinical implementation. Additionally, the wide confidence interval of the AUC in the internal validation set (0.699–1.000) underscores the instability of performance estimates due to the small event count, further emphasizing the need for larger-scale validation studies. The included variables were primarily static baseline data and did not encompass key dynamic factors like feeding practices or genetic and environmental influences. Furthermore, although we ensured that predictors preceded the outcome in time, the cross-sectional nature of the outcome assessment (single time point at 12 months) means that the model does not capture dynamic changes in growth status over time. Future studies should incorporate longitudinal outcome measurements. Additionally, the use of 24-hour dietary recall for assessing complementary food diversity and intake may introduce recall bias, despite our efforts to standardize data collection. The model’s applicability to special populations, such as preterm infants, was not evaluated and requires dedicated future research. Furthermore, before clinical implementation, prospective validation in independent cohorts is necessary. Cost-effectiveness analyses comparing this model to standard growth monitoring should be conducted. Future work will also focus on developing a user-friendly mobile application or integrating the nomogram into electronic health record systems to facilitate real-time risk assessment in primary care settings. Additionally, this study did not stratify infants by age in months (e.g., 0–6, 6–9, 9–12 months), despite known differences in recommended dietary allowances for protein, iron, and other nutrients across these age windows. The model assumes a uniform relationship between predictors and stunting at 12 months across the entire 0–12 month range, which may not hold. Future studies with larger sample sizes should perform age-stratified analyses and incorporate age-specific nutrient intake standards.

## Conclusion

In conclusion, the novel nutritional scoring system and gradient boosting prediction algorithm developed in this study demonstrate good performance for the identification of infant stunting at 12 months. The nomogram tool provides a practical aid for clinical risk assessment. The findings clarify the mechanistic roles and effect strengths of core influencing factors such as length growth velocity and hemoglobin, offering a quantitative reference for child healthcare physicians to develop targeted nutritional intervention strategies.

## Data Availability

The original contributions presented in the study are included in the article/Supplementary Material, further inquiries can be directed to the corresponding author.

## Supplementary material

Supplementary Figure 1 SHAP Feature Importance Plot (A) and Dependence Plot (B). X1: Infant Weight-for-age Z-score; X2: Infant Length-for-age Z-score; X3: Length growth velocity; X4: Number of Complementary Food Types; X5: Infant Hb.

Supplementary Table 1 Variable assignment table for multivariate logistic regression and LASSO regression.

## References

[1] Benjamin-Chung J Mertens A Colford JM Jr Coyle J van der Laan MJ Hubbard AE. Causes and consequences of child growth faltering in low-resource settings. Nature. (2023) 621(7979):568–76. 10.1038/s41586-023-06501-x 37704722 PMC10511328

[2] Allotey J Archer L Coomar D Snell KI Smuk M Oakey L. Development and validation of prediction models for fetal growth restriction and birthweight: an individual participant data meta-analysis. Health Technol Assess. (2024) 28(47):1–119. 10.3310/DABW4814 PMC11404361 39252507

[3] Li Ching Ng L Patel S Plourde H Besner ME Lapointe A Bizgu V. The association between BMI trajectories and bronchopulmonary dysplasia among very preterm infants. Pediatr Res. (2023) 93(6):1609–15. 10.1038/s41390-022-02358-4 36414708

[4] Cooke R Goulet O Huysentruyt K Joosten K Khadilkar AV Mao M. Catch-up growth in infants and young children with faltering growth: expert opinion to guide general clinicians. J Pediatr Gastroenterol Nutr. (2023) 77(1):7–15. 10.1097/MPG.0000000000003784 36976274 PMC10259217

[5] Saha J Chouhan P Malik NI Ghosh T Das P Shahid M. Effects of dietary diversity on growth outcomes of children aged 6 to 23 months in India: evidence from national family and health survey. Nutrients. (2023) 15(1):159. 10.3390/nu15010159 PMC9824371 36615816

[6] Mengistu YG Hailemariam D Roro MA Endris BS Tesfamariam K Gebreyesus SH. Intrauterine growth pattern in butajira HDSS, southern Ethiopia: bUNMAP pregnancy cohort. BMC Pediatr. (2023) 23(1):422. 10.1186/s12887-023-04244-2 37620778 PMC10464298

[7] Kakatsaki I Papanikolaou S Roumeliotaki T Anagnostatou NH Lygerou I Hatzidaki E. The prevalence of small for gestational age and extrauterine growth restriction among extremely and very preterm neonates, using different growth curves, and its association with clinical and nutritional factors. Nutrients. (2023) 15(15):3290. 10.3390/nu15153290 37571226 PMC10420820

[8] Ahmed SM Brintz BJ Pavlinac PB Shahrin L Huq S Levine AC. Derivation and external validation of clinical prediction rules identifying children at risk of linear growth faltering. eLife. (2023) 12:e78491. 10.7554/eLife.78491 36607225 PMC9833824

[9] Ogwel B Mzazi VH Oreso C Anyango RO Oreso C Ochieng JB. Predictive modelling of linear growth faltering among pediatric patients with diarrhea in rural western Kenya: an explainable machine learning approach. BMC Med Inform Decis Mak. (2024) 24(1):368. 10.1186/s12911-024-02779-7 39623435 PMC11613762

[10] Chen RY Mostafa I Hibberd MC Das S Mahfuz M Naila NN. A Microbiota-directed food intervention for undernourished children. N Engl J Med. (2021) 384(16):1517–28. 10.1056/NEJMoa2023294 33826814 PMC7993600

[11] Calek E Binder J Palmrich P Eibensteiner F Thajer A Kainz T. Effects of intrauterine growth restriction (IUGR) on growth and body composition compared to constitutionally small infant. Nutrients. (2023) 15(19):4158. 10.3390/nu15194158 37836441 PMC10574227

[12] Noll ATR van Hoogstraten A Nulens K van Geloven N Van Mieghem T Shinar S. Outcome of monochorionic diamniotic twin pregnancy with selective fetal growth restriction and continuous or intermittent absent or reversed end-diastolic umbilical artery flow: international multicenter cohort study. Ultrasound Obstet Gynecol. (2025) 66(1):41–50. 10.1002/uog.29241 40443107 PMC12209694

[13] Garcia-Manau P Bonacina E Martin-Alonso R Martin L Palacios A Sanchez-Camps ML. Angiogenic factors versus fetomaternal Doppler for fetal growth restriction at term: an open-label, randomized controlled trial. Nat Med. (2025) 31(3):1008–15. 10.1038/s41591-024-03421-9 39775039

[14] Fenton TR Gilbert N Elmrayed S Fenton CJ Boctor DL. What is normal growth? Principles, practicalities and pitfalls of growth assessments in infants and children. Ann Nutr Metab. (2024) 80(Suppl 1):7–17. 10.1159/000541226 39602909

[15] Bonell A Vicedo-Cabrera AM Moirano G Sonko B Jeffries D Moore SE. Effect of heat stress in the first 1000 days of life on fetal and infant growth: a secondary analysis of the ENID randomised controlled trial. Lancet Planet Health. (2024) 8(10):e734–43. 10.1016/S2542-5196(24)00208-0 39393375 PMC11462510

[16] Shoji H Murano Y Saitoh Y Ikeda N Ohkawa N Nishizaki N. Use of head and chest circumference ratio as an Index of fetal growth retardation in preterm infants. Nutrients. (2022) 14(22):4942. 10.3390/nu14224942 36432628 PMC9694309

[17] Larsen ML Krebs L Hoei-Hansen CE Kumar S. Assessment of fetal growth trajectory identifies infants at high risk of perinatal mortality. Ultrasound Obstet Gynecol. (2024) 63(6):764–71. 10.1002/uog.27610 38339783

[18] Wang YS Shen W Wu F Mao J Liu L Chang YM. Factors influencing extrauterine growth retardation in singleton-non-small for gestational age infants in China: a prospective multicenter study. Pediatr Neonatol. (2022) 63(6):590–8. 10.1016/j.pedneo.2022.04.013 36241604

[19] van Klink JMM van Zwet EW Lopriore E Roest AAW Haak MC Slaghekke F. Does catch-up growth Come with a cognitive cost? Cognitive outcome and growth patterns in growth discordant identical twins. J Pediatr. (2024) 275:114223. 10.1016/j.jpeds.2024.114223 39097263

[20] Stevenson NJ Lai MM Starkman HE Colditz PB Wixey JA. Electroencephalographic studies in growth-restricted and small-for-gestational-age neonates. Pediatr Res. (2022) 92(6):1527–34. 10.1038/s41390-022-01992-2 35197567 PMC9771813

[21] Reforma LG Febres-Cordero D Trochtenberg A Modest AM Collier AY Spiel MH. Incidence of small-for-gestational-age infant birthweight following early intertwin fetal growth discordance in dichorionic and monochorionic twin pregnancies. Am J Obstet Gynecol. (2022) 226(5):726.e721–729. 10.1016/j.ajog.2021.11.1358 PMC9064885 34838799

[22] Busiah K Roda C Crosnier AS Brassier A Servais A Wicker C. Pubertal origin of growth retardation in inborn errors of protein metabolism: a longitudinal cohort study. Mol Genet Metab. (2024) 141(3):108123. 10.1016/j.ymgme.2023.108123 38219674

[23] Bahri Khomami M Hashemi S Shorakae S Harrison CL Piltonen TT Romualdi D. Systematic review and meta-analysis of birth outcomes in women with polycystic ovary syndrome. Nat Commun. (2024) 15(1):5592. 10.1038/s41467-024-49752-6 38965241 PMC11224419

[24] Gao L Shen W Wu F Mao J Liu L Chang YM. Real-time predictive model of extrauterine growth retardation in preterm infants with gestational age less than 32 weeks. Sci Rep. (2024) 14(1):12884. 10.1038/s41598-024-63593-9 38839838 PMC11153599

