# Supplementary material for: A novel nutritional tool to identify infants at risk of stunting
Source: Front Pediatr. 2026 Jun 8;14:1782208. doi: 10.3389/fped.2026.1782208 (PMC13283820; doi:10.3389/fped.2026.1782208)
Supplement: Supplementary Figure 1 — SHAP Feature Importance Plot (A) and Dependence Plot (B). X1: Infant Weight-for-age Z-score; X2: Infant Length-for-age Z-score; X3: Length growth velocity; X4: Number of Complementary Food Types; X5: Infant Hb. [file Supplementaryfile1.docx]

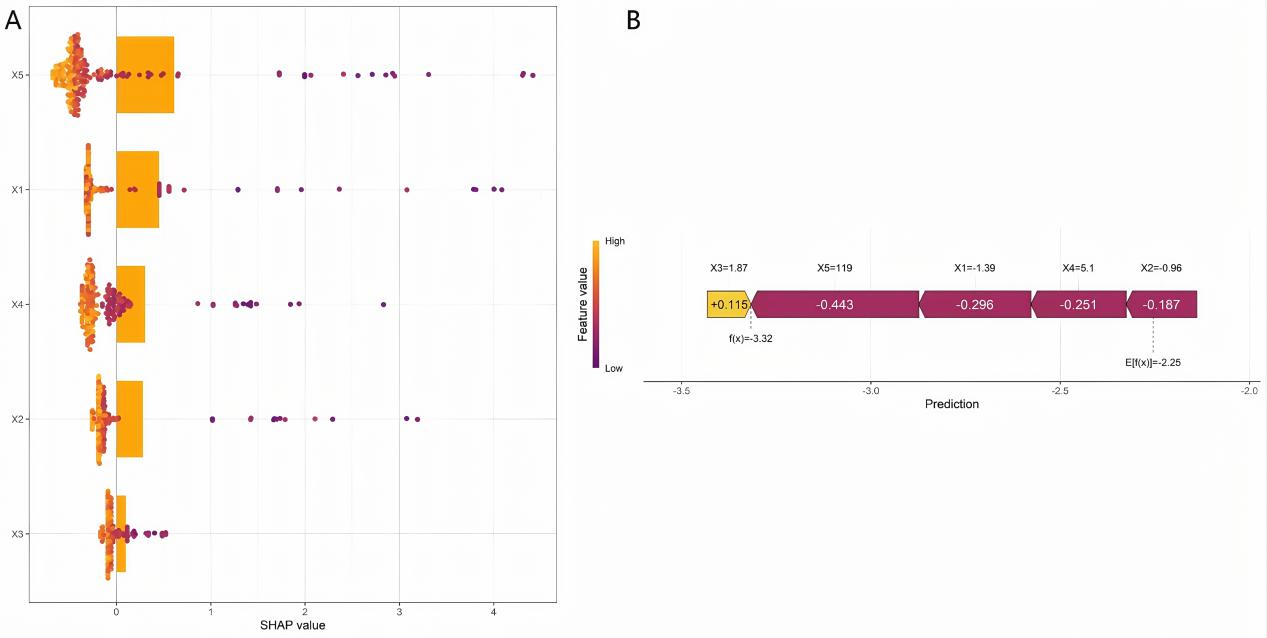


**Supplemental Figure 1.** SHAP Feature Importance Plot (A) and Dependence Plot (B). **Note:** X1: Infant Weight-for-age Z-score; X2: Infant Length-for-age Z-score; X3: Length growth velocity; X4: Number of Complementary Food Types; X5: Infant Hb
