# Supplementary material for: A novel nutritional tool to identify infants at risk of stunting
Source: Front Pediatr. 2026 Jun 8;14:1782208. doi: 10.3389/fped.2026.1782208 (PMC13283820; doi:10.3389/fped.2026.1782208)
Supplement: Supplementary Table 1 — Variable assignment table for multivariate logistic regression and LASSO regression. [file Table1.docx]

**Supplemental Table 1.** Variable Assignment Table.

| Variable | Meaning | Assignment |
| --- | --- | --- |
| X1 | Infant Weight-for-age Z-score | Continuous variable |
| X2 | Infant Length-for-age Z-score | Continuous variable |
| X3 | Length growth velocity | Continuous variable |
| X4 | Number of Complementary Food Types | Continuous variable |
| X5 | Infant Hb | Continuous variable |
| Y | Growth retardation status | 1=Growth retardation group, 0=Normal group |
